# The difficulty of oral speech act production tasks in second language pragmatics testing

**DOI:** 10.3389/fpsyg.2023.1096399

**Published:** 2023-02-03

**Authors:** Weiying Huang, Xiaofei Lu

**Affiliations:** ^1^School of Foreign Languages, East China University of Technology, Nanchang, China; ^2^Department of Applied Linguistics, The Pennsylvania State University, University Park, PA, United States

**Keywords:** pragmatic ability, speech acts, situational variables, task difficulty estimates, L2 pragmatics testing

## Abstract

This study examined the relative difficulty of oral speech act production tasks involving eight different types of speech acts for Chinese English as a foreign language (EFL) learners and the effects of three contextual variables, namely, power, social distance, and imposition, on such difficulty. Eight Oral Discourse Completion Task items, each representing a unique combination of the three contextual variables, were designed for each speech act. Eighty Chinese EFL learners responded to these items and their responses were rated for appropriateness by two native-speaking college English instructors. A Many-facet Rasch Measurement analysis suggested that the eight speech acts can be ordered by ascending difficulty as follows: Thank, Request, Suggestion, Disagreement, Invitation, Refusal, Offer, and Apology. Significant effects on performance scores were found for the interaction between each of the three contextual variables and speech act, and the specific effects observed varied by speech act. The implications of our findings for L2 pragmatics testing are discussed.

## Introduction

Pragmatic ability, that is, the ability to understand the intended meanings communicated by the speaker and to use language appropriately in various communicative contexts (Ross and Kasper, 2013; Ren, 2022), is a crucial component in models of communicative language ability (Purpura, 2004; Bachman and Palmer, 2010). Albeit recent developments in second language pragmatics testing have shown a growing interest in interactive, discursively oriented assessment of interactional competence (for instance, Grabowski, 2009, 2013; Youn, 2015, 2019; Ikeda, 2017; Galaczi and Taylor, 2018), an important part of second language (L2) pragmatics testing involves assessing L2 learners’ ability to realize different speech acts under different circumstances (Ross and Kasper, 2013). Research in this area has attended to the effects of different task features and contextual variables on the difficulty of pragmatic tasks (e.g., Hudson, 2001; Taguchi, 2007; Youn, 2019). At the same time, while language users’ ability to perform various speech acts has been recognized as the universality of pragmatics (Searle, 1969), linguistic means to engage in those speech acts and the socio-pragmatic norms associated with them exhibit considerable variation across languages and cultures (Taguchi, 2012). This variation poses challenges for learning L2 speech acts and points to the need to take first language (L1) cultural background into account in assessing task difficulty. As identified in Roever’s (2007) study, one fourth of his test items in a pragmatics test showed differential functioning for test takers of Asian and European background. Indeed, a few studies have designed or evaluated L2 pragmatics tests with learners’ L1 background in mind (e.g., Fulcher and Reiter, 2003; Liu, 2006, 2007). However, systematical explorations of the difficulty of L2 oral production tasks involving a diverse range of speech acts and representing diverse combinations of contextual factors for learners from a specific L1 cultural background remain scant.

### Task difficulty in oral proficiency assessment

Commonly used frameworks of task difficulty within second language acquisition (SLA) have focused on analyzing the degree of cognitive load and complexity of tasks (e.g., Skehan, 1998; Robinson, 2001). Skehan’s (1998) Limited Attentional Capacity Model and Robinson’s (2001) Cognition Hypothesis both hypothesize that manipulating the cognitive complexity and communicative requirements of a task will produce differential cognitive and communicative demands and affect the accuracy and complexity of the language that learners use to perform the task. Skehan (1998) proposed three dimensions of task difficulty: code complexity (i.e., the variety and difficulty of the linguistic forms required for performing the task), cognitive complexity (i.e., the cognitive processing demands of the task content, such as the type of information to be processed), and communicative stress (i.e., stress caused by task-related factors such as time pressure). His model predicts a competition between accuracy and complexity as a result of limited attentional resources. Robinson’s (2001) triadic framework distinguishes task complexity features affected by cognitive factors (e.g., number of elements to deal with) from task condition features affected by interactional factors (e.g., power difference of the interlocutors) and task difficulty features affected by learner factors (e.g., learner motivation). His Cognition Hypothesis claims that increased task complexity may simultaneously promote linguistic complexity and accuracy as learners will activate and allocate more attentional resources to handle the higher cognitive load.

A few language assessment studies have applied these cognitive models of task complexity to examine the effect of varying task conditions on task difficulty in speaking tests. Based on Skehan’s (1998) cognitive complexity framework, Iwashita et al. (2001) manipulated the performance conditions of a series of picture-based narrative task in terms of perspective (first vs. third person perspective), immediacy (here and now vs. there and then), adequacy (a complete set of pictures vs. an incomplete set), and planning time (no planning time vs. 3 min planning time). They found no significant effect of the varying performance conditions on either the test-takers’ discourse in terms of fluency, complexity, or accuracy or the quality ratings of their performance. Elder et al. (2002) further reported that the varying performance conditions did not affect task difficulty as perceived by the test-takers. They concluded that their results did not support Skehan’s framework in the case of oral proficiency assessment. The lack of score sensitivity to varying task conditions in speaking tests has also been reported in other studies (Fulcher, 1996; Fulcher and Reiter, 2003). Accordingly, Fulcher and Reiter (2003) suggested that L2 pragmatics test designers “may look to pragmatic categories and cultural factors to develop task types” (p. 339).

### Speech acts, contextual variables, and task difficulty in L2 pragmatics testing

A common way to attend to pragmatic categories in L2 pragmatics testing has been to look at different speech acts. Indeed, the speech act paradigm has played an important role in pragmatics testing since the 1980s, with the influence of studies in the Cross-Cultural Speech Act Realization Patterns (CCSARP) project initiated to investigate cross-cultural variations in speech act realization (Cohen and Olshtain, 1981; Blum-Kulka et al., 1989). Given that the linguistic realization patterns of speech acts have been found to differ from culture to culture (Gass and Neu, 1996; Taguchi, 2012), L2 learners’ pragmatic ability to realize different speech acts in the target language has been recognized as an essential component of their L2 communicative language ability (Bachman, 1990; Bachman and Palmer, 1996, 2010) and a prominent target construct of L2 pragmatics testing (Roever, 2011).

Pragmatics tests of speech act realization have drawn heavily from Speech Act theory (Searle, 1969) and Politeness theory (Brown and Levinson, 1987). Speech Act theory views as the minimum unit of human communication the performance of different acts through language (e.g., apology and refusal) and distinguishes direct speech acts, where the speaker directly states the intended meaning, usually with certain conventionalized linguistic forms, from indirect ones, where the speaker says more than or something other than the intended meaning (Searle, 1975). In Politeness theory, the directness of speech acts is seen to vary systematically with three contextual properties defined *a priori*, i.e., power, social distance, and rank of imposition (Brown and Levinson, 1987). L2 pragmatics tests commonly examine L2 learners’ realization of different speech acts in situations with different contextual properties, although the most commonly investigated types of speech acts have centered around apology, refusal, and request (Hudson et al., 1992, 1995; Yamashita, 1996; Yoshitake, 1997; Ahn, 2005; Roever, 2005, 2006; Liu, 2006, 2007).

Among the task types used to test speech act production in pragmatics testing, Discourse Completion Tasks (DCTs) are used more widely than other types such as role plays and sociopragmatic judgment tasks (Martínez-Flor and Usó-Juan, 2010). Although DCTs are artificial in nature (Brown, 2001; Golato, 2003), they allow for the evaluation of learners’ pragmatic knowledge and are the most prevalent data collection method in L2 pragmatics. Hudson et al. (1992, 1995) designed a prototypical pragmatics test battery for apology, refusal, and request, which included six types of DCTs, namely, Written Discourse Completion Tasks (WDCT), Multiple-Choice Discourse Completion Tasks (MDCT), Oral Discourse Completion Tasks (ODCTs), Discourse Role-Play Tasks (DRPT), Discourse Self-Assessment Tasks (DSAT), and Role-Play Self-assessments (RPSA). All tasks other than self-assessments were designed around high/low settings of power, social distance, and imposition (Brown and Levinson, 1987), rendering eight combinations of these contextual variables. Each task required test-takers to produce an oral or written response to a specific scenario representing a particular combination of contextual variables.

A limited number of studies have examined how pragmatic production tasks involving different speech acts compared with each other in terms of difficulty or how different contextual variables affect the difficulty of such tasks, sometimes with attention to the effects of assessment methods and/or L1 cultural background. Hudson (2001) examined the effects of three assessment methods (i.e., WDCTs, language lab DCTs, and role-play scenarios) and three contextual variables (i.e., power, social distance, and imposition) on the scores assigned to pragmatic productions tasks involving three speech acts (i.e., apologies, refusals, and requests) among Japanese English as a second language (ESL) learners. He found that lab DCTs were slightly more difficult than the other two methods and that apologies were rated slightly higher than refusals and requests. He reported minimal effects of the contextual variables on the scores, with only imposition showing a slight effect, and attributed the lack of effects to the homogeneity of the participants’ proficiency level. Fulcher and Reiter (2003) examined how social power and imposition as well as their interaction with learners’ L1 background affect test-takers’ pragmatic performance. Six role-play tasks representing six combinations of the two contextual variables were used to elicit L2 English learners’ realization of request. Significant effects were found for both contextual variables, the two-way interaction between social power and L1 background, and the three-way interaction between social power, imposition and L1 background. Roever (2004) reviewed item difficulty in pragmatics tests including learners’ interpretation of routines, implicature and production of speech acts and identified degree of imposition as a source of speech act difficulty. The effect of degree of imposition on the difficulty of speech act performance was also evident in Taguchi’s (2007) study, in which she examined the effects of task difficulty on Japanese EFL learners’ oral production of requests and refusals. She operationalized task difficulty as two situation types, one with an equal power relationship, small social distance, and a small degree of imposition (PDR-low), and the other with greater power for the listener, large social distance, and a large degree of imposition (PDR-high). She reported that L2 learners produced speech acts significantly more easily and quickly in the PDR-low situation than in the PDR-high situation. In a study designed to evaluate the reliability of three test methods (WDCT, MDCT, and DST) for assessing the pragmatic knowledge of Chinese EFL learners, Liu (2006) reported that the three methods were reasonably reliable, and that the apology subtest proved consistently more difficult than the request subtest across three test methods. However, compliment responses and refusals were found relatively easy while requests were more difficult for L2 Chinese learners in Li et al. (2019). Krish and May (2020) identified interference of L1 cultural knowledge and linguistic rules in L2 Chinese learners’ pragmatic performance of five speech acts: compliments, requests, refusals, apologies, and complaints.

Taken together, these studies have provided evidence that pragmatic tasks involving different speech acts may have varying degrees of difficulty for L2 learners and that their relative difficulty may be affected by the learners’ L1 background and proficiency level, the assessment method used, and the contextual variables of power, social distance, and imposition. Meanwhile, it can also be seen that the range of speech acts and the range of combinations of different contextual variables that have been investigated in previous studies were both small, and the interaction between the contextual variables and speech acts has been underexamined. How learners’ native culture may influence their performance in pragmatics tests has barely been touched upon.

### Objectives

The current study contributes to the limited body of research in this area by examining the difficulty of oral production tasks involving different types of speech acts for Chinese English as foreign language (EFL) learners. In response to the call for broadening the range of pragmatic tasks and attending to the effects of relevant contextual variables in assessing task difficulty in pragmatics testing (Taguchi, 2007; Youn, 2019), we include eight speech acts and three contextual variables in designing the oral production tasks. It is our hope that our analysis will provide useful insight into the relative difficulty of oral production tasks involving different speech acts for Chinese EFL learners and the effects of the interaction between the contextual variables and speech act on task difficulty in L2 pragmatics tests. Informed by findings of previous studies, we explored these issues with a single assessment method and a group of learners from a single L1 background (i.e., Chinese EFL learners) representing diverse proficiency levels.

### Research questions

The present study explores the difficulty of oral speech act production tasks for Chinese EFL learners in L2 pragmatics testing by addressing the following research questions:

1. What is the order of the difficulty estimates for oral speech act production tasks involving the speech acts of Apology, Disagreement, Thank, Request, Suggestion, Invitation, Offer and Refusal?
2. How do social distance, relative power, and imposition interact with speech act to affect the difficulty of oral speech act production tasks?

## Methodology

### Participants

Eighty Chinese EFL learners (24 male, 56 female) with an average age of 20.6 from three universities in south China responded to an open call to participate in the current study. The participants represented a range of disciplinary backgrounds, years in college, and language proficiency levels, with 35 first-and second-year non-English major undergraduate students from various arts and science disciplines, 40 first-and third-year English major undergraduate students, and five applied linguistics postgraduate students who majored in English in college. No participant had been abroad for over 1 month.

### Instruments

Given that our participants were all undergraduate and postgraduate students, we decided to test their pragmatic performance on speech acts commonly used in university settings. To this end, we identified 20 speech acts commonly discussed in the Interlanguage Pragmatics (ILP) literature and invited 28 L1 English American college students to rate the frequency of using each of them in their university life on a five-point scale. Based on their ratings, we included the following eight highest ranked speech acts in the current study: Apology, Disagreement, Thank, Request, Suggestion, Invitation, Offer, and Refusal.

We elicited the participants’ performance in producing target speech acts orally using Oral Discourse Completion Tasks (ODCTs). DCTs have been criticized for limited generalizability (Roever, 2011), but ODCTs can measure online performance under time pressure (Roever, 2004), which improves their authenticity and generalizability. To test the participants’ pragmatic ability to cope with different contexts, we incorporated different combinations of three contextual variables, i.e., relative power, social distance, and imposition in the ODCTs, with the values of these variables specified for each speech act production task. Relative power (P) refers to the power of the speaker with respect to the hearer (Brown and Levinson, 1987), and P+, P−, and P= denote the speaker has more, less, or equal power relative to the hearer, respectively, with more power defined as a higher rank, title, or social position or greater control of the assets in the situation. We excluded scenarios with the P+ feature in the current study as we limited the discourse context to the university setting, in which such scenarios were uncommon for our participants. Common scenarios with the P= feature included talking to classmates and roommates, and common scenarios with the P− feature included talking to faculty and staff members. Social Distance (D) refers to the degree of familiarity and solidarity between the speaker and the hearer (Brown and Levinson, 1987). D+ indicates that the speaker and hearer are unfamiliar with each other, and D-indicates that they are familiar with each other. Imposition (R) refers to the expenditure of goods and/or services by the hearer or the obligation of the speaker to perform an act (Brown and Levinson, 1987). Given that the nature of this variable varies with different speech acts, we determined the value of this variable for each item in two steps. The speech events in the ODCTs were first ranked for imposition by two native speaker consultants through collaborative discussion. The rankings were then used to code the task items pertaining to the same speech act as either R+ (high imposition) or R− (low imposition), depending on whether each item was ranked in the top or bottom half among the items for that speech act.

We initially developed eight ODCT items for each target speech act, each with a scenario reflecting a unique combination of the three contextual variables, as summarized in Table 1. Each item was checked by two native speaker consultants for authenticity. The consultants recommended the removal of four items for Disagreement on the basis that they represented unrealistic scenarios. One consultant indicated that “it’s better to remain quiet if you do not agree in these cases.” Therefore, only four items were retained for Disagreement (Item 1, 2, 3, 5). All other items were accepted by the consultants as authentic. The final test battery thus consisted of 60 ODCT items (see Appendix).

**Table 1:** Combinations of the three contextual variables represented by the eight ODCT items for each speech act.

	Item 1	Item 2	Item 3	Item 4	Item 5	Item 6	Item 7	Item 8
D	−	+	−	+	−	+	−	+
P	=	=	−	−	=	=	−	−
R	−	−	−	−	+	+	+	+

D, social distance; P, relative power; R, imposition.

### Procedure

The pragmatics test was first piloted with five Chinese EFL learners enrolled in the same university who did not participate in the actual study. They all found the scenario descriptions clear, but two participants identified several words in the descriptions that caused some comprehension difficulties. We thus added Chinese glosses to those words to minimize potential comprehension problems. Based on the maximum time they took to complete each item, we set the time limit to 20 s for the first 50 items and 50 s for the last 10 items due to the extended length of these items.

The final test was administered to the 80 participants in a large language lab in 12 groups of six to seven, with ample space between any two participants to minimize interference from each other. At the beginning of each session, one researcher provided instructions in English, illustrated the scenario descriptions and the types of oral response expected with an example, and confirmed that all participants understood the instructions and requirements. The researcher then presented the scenario descriptions and their corresponding time limits using PowerPoint slides on a screen in the front of the lab one by one. There was a signal for the participants to stop speaking at the end of the time limit for each item, and the next slide was shown. The entire session lasted about 1 h for each group. Each participant’s responses were recorded by the computer and then saved in a separate audio file for rating and further analysis.

### Data analysis

Each participant’s oral response to each item was firstly transcribed and their written responses were independently rated for pragmatic appropriateness by two native speakers of American English, both of whom were experienced English instructors at the university. A holistic five-point scale was adopted from the five-level rating scale constructed to evaluate Chinese EFL’s written speech act performance by Chen and Liu (2016). Inter-rater reliability, assessed using Spearman’s rank correlation, reached 0.823 (*p* < 0.001). The final score of each response was the mean of the two scores, and the overall test score of each participant was the sum of the scores for all responses by that participant.

We subjected the scores of the 80 participants’ responses to the 60 ODCT items to a Many-facet Rasch Measurement (MFRM) analysis within Item Response Theory (McNamara and Knoch, 2012) using the FACETS 3.71.3 (Linacre, 2013) for the analyses, with participants, speech acts, and item types as facets to assess the difficulty of items for each speech act as well as items of each of the eight types representing a specific combination of the three contextual variables. We further performed a series of two-way ANOVAs, each with speech act and one of the three contextual variables as independent variables and participants’ response scores as the dependent variable, to examine the effects of the interaction between each contextual variable and speech act on the difficulty of oral speech act production tasks. Cohen’s *D*, or standardized mean difference, was adopted as an effect size measure. Following Cohen (1969), we characterized effect sizes as small, medium, and large if the η_p_^2^ values were larger than 0.0099, 0.0588, and 0.1379, respectively.

## Results

### Research question 1: Order of the difficulty estimates for tasks involving different speech tasks

**Figure 1:**
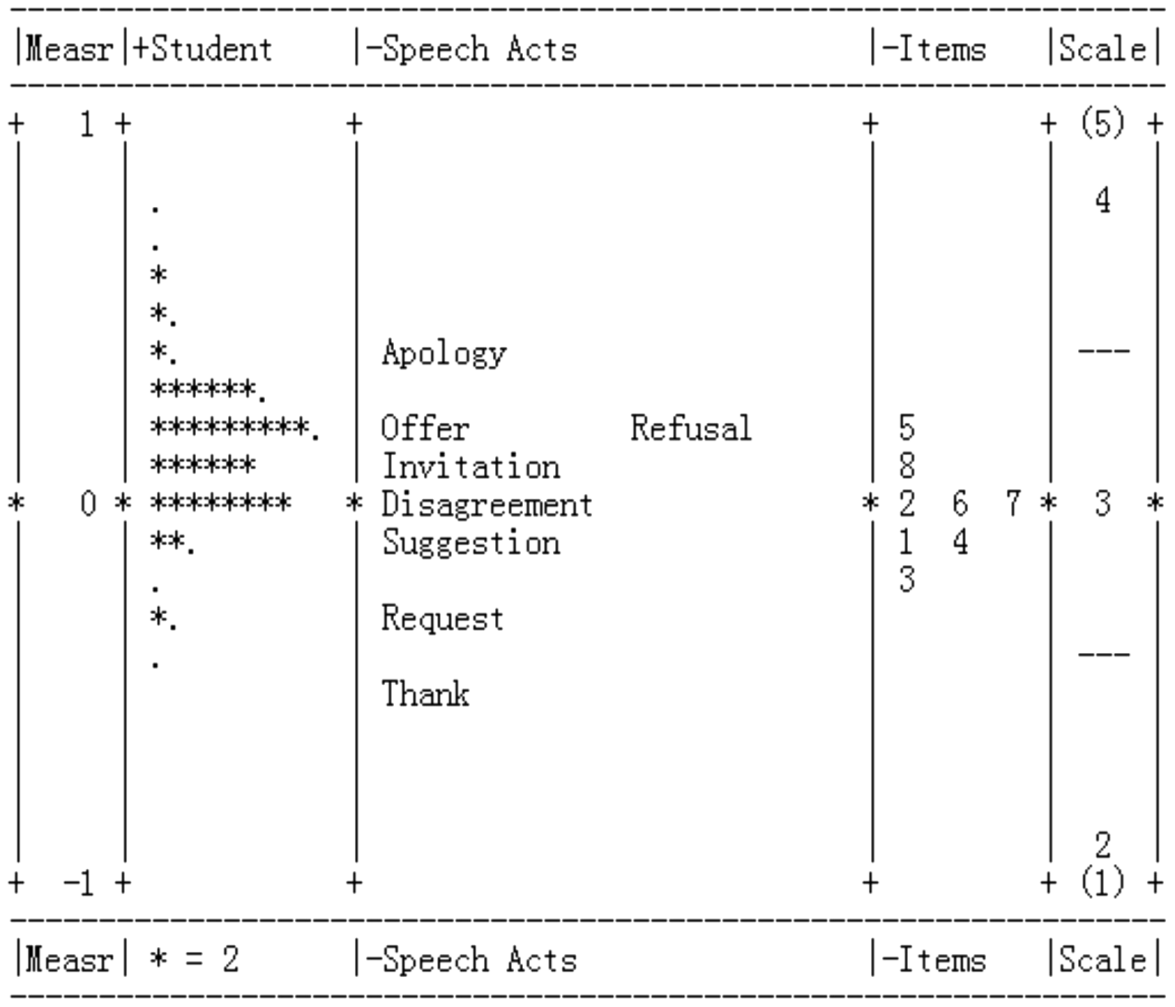
Results of the Many-facet Rasch Measurement analysis of participant performance.

The MFRM analysis placed the estimates of the three facets (i.e., participants, speech acts, and item types) on a single measurement scale, as shown in Figure 1. The range of the measurements was within two logits, likely due to the narrow range of the ILP competence of our participants. The average person measure was 0.16, with a standard deviation of 0.22. Only four misfitting persons were identified with Z scores larger than two.

For the speech act measures, the mean measure was set at zero and the standard deviation was calculated to be 0.30. Thank and Request were found to be the easiest, followed by Suggestion, Disagreement, and Invitation. Refusal, Offer, and Apology were found to be the most difficult among the eight speech acts.

Facets also generates an overall estimate of the extent to which items are at reliably different levels of difficulty. The reliability of separation index denotes the reliability with which the items included in the analysis are separated (i.e., how different the item difficulty measures are), and the fixed chi-square test for the items tests the hypothesis that all items are of the same level of difficulty, after accounting for measurement error. The reliability of separation was reported as 0.90 [χ^2^(7) = 74.0, *p* = 0.000], indicating significant differences among the test items in terms of difficulty.

For the item type measures, the mean measure was set at zero and the standard deviation was calculated to be 0.11, indicating a low range of difficulty. Item 3 (D−, P−, and R−) was the easiest item type, followed by items 1 (D−, P=, R−) and 4 (D+, P−, R−). Item 5 (D−, P=, R+) was the most difficult item type, followed by item 8 (D+, P−, R+). These results suggest that items with lower imposition (R−) tended to be easier than those with higher imposition (R+).

To sum up, the MFRM analysis results suggested that the eight speech acts can be ordered by ascending difficulty as follows: Thank, Request, Suggestion, Disagreement, Invitation, Refusal, Offer, and Apology. The results also suggested a potential effect of imposition on learners’ oral speech act production performance.

### Research question 2: Effects of the interaction between each of the three contextual variables and speech act on the difficulty of oral speech act production tasks

Three separate two-way ANOVAs were conducted to investigate the effects of the interaction between each contextual factor and speech act on the difficulty of oral speech act production tasks. The four items for Disagreement were excluded from these analyses because not all values for all three variables were represented among these items as a result of the removal of four Disagreement items. The Levene test indicated that the assumption of equal variance across groups was violated (*p* < 0.05). However, the ANOVA *F* test has been shown to be robust if the sample is large, the group sizes are equal, and the largest group standard deviation is not larger than twice the smallest group standard deviation (e.g., Agresti et al., 2017). Given that our dataset met these criteria, we proceeded with the two-way ANOVAs followed by pairwise comparisons using the Tamhane’s T2 *post hoc* test, which does not assume equal variances across groups.

### Social distance

**Figure 2:**
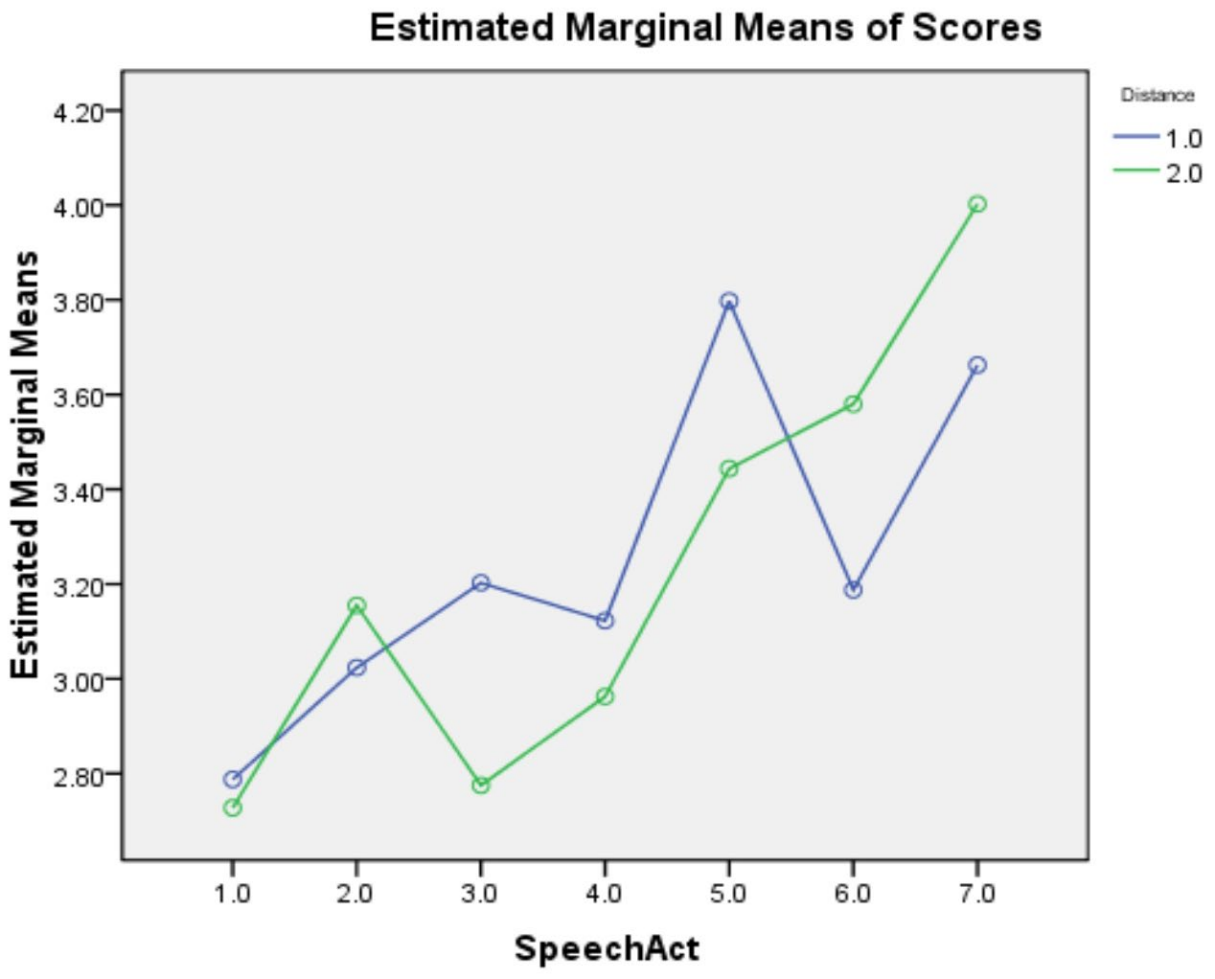
Profile plots for the interaction between speech act and social distance. Speech act codes: 1 = Apology; 2 = Invitation; 3 = Offer; 4 = Refusal; 5 = Request; 6 = Suggestion; 7 = Thank. Social distance codes: 1 = D−; 2 = D+.

As shown in Table 2, the main effect of speech act was statistically significant with a large effect size [*F*(6,153) = 68.243, *p* = 0.000, η_p_^2^ = 0.270], but the main effect of social distance was insignificant [*F*(1,158) = 0.316, *p* = 0.574, η_p_^2^ = 000]. The interaction effect between the two factors was significant with a medium effect size [*F*(1,158) = 12.127, *p* = 0.000, η_p_^2^ = 0.062]. Pairwise comparisons revealed that, compared to items with the D+ feature, those with the D-feature were significantly easier for Offer and Request but significantly harder for Suggestion and Thank. These results are also visualized in Figure 2.

**Table 2:** Comparison of mean task performance by speech act and social distance.

Speech act	*N*	Mean/SD		Pairwise comparisons	Analysis of variance				
		D−	D+	*p*		df	*F*	*p*	η_p_^2^
Apology	80	2.788/0.63	2.728/0.73	0.581	Speech act	6	68.243	0.000	0.270
Invitation	80	3.024/0.51	3.155/0.53	0.114	Social distance	1	0.316	0.574	0.000
Offer	80	3.203/0.59	2.775/0.67	0.000	Interaction	6	12.127	0.000	0.062
Refusal	80	3.123/0.48	2.963/0.65	0.080					
Request	80	3.798/0.57	3.444/0.50	0.000					
Suggestion	80	3.188/0.49	3.580/0.54	0.000					
Thank	80	3.662/0.57	4.003/0.61	0.000					

### Power

**Figure 3:**
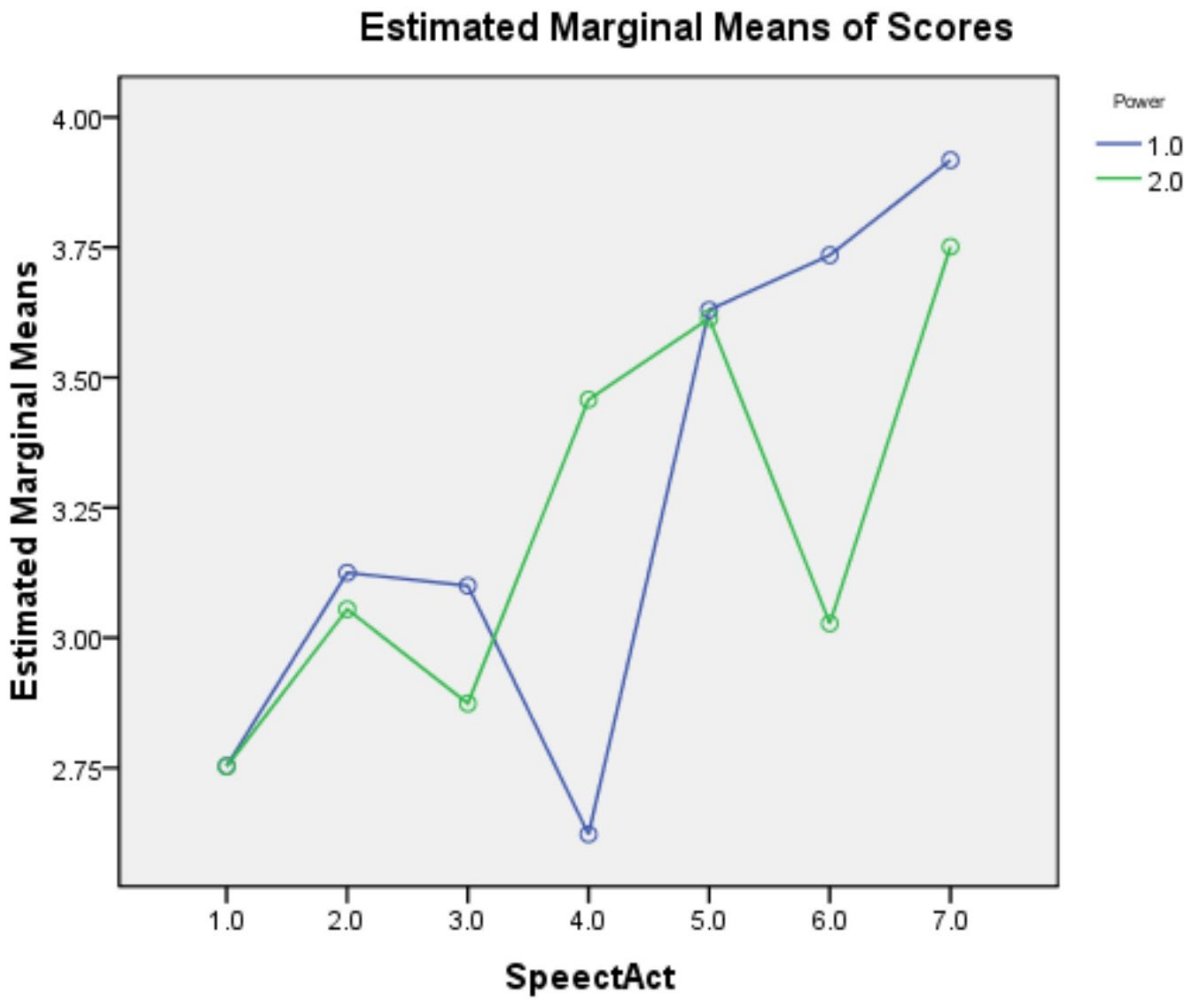
Profile plots for the interaction between speech act and power. Speech act codes: 1 = Apology; 2 = Invitation; 3 = Offer; 4 = Refusal; 5 = Request; 6 = Suggestion; 7 = Thank. Power codes: 1 = P−; 2 = *p* = .

As shown in Table 3, the main effect of speech act was statistically significant with a large effect size [*F*(6,153) = 65.843, *p* = 0.000, η_p_^2^ = 0.263], but the main effect of power was insignificant [*F*(1,158) = 1.986, *p* = 0.159, η_p_^2^ = 0.002]. The interaction effect between the two factors was significant with a medium effect size [*F*(1,158) = 23.575, *p* = 0.000, η_p_^2^ = 0.113]. Pairwise comparisons revealed that, compared with items with the P= feature, those with the P-feature were significantly easier for Offer and Suggestion but significantly harder for Refusal. These results are also visualized in Figure 3.

**Table 3:** Comparison of mean task performance by speech act and power.

Speech act	*N*	Mean/SD		Pairwise comparisons	Analysis of variance				
		P−	P=	*p*		df	*F*	*p*	η_p_^2^
Apology	80	2.753/0.66	2.752/0.72	0.991	Speech act	6	65.843	0.000	0.263
Invitation	80	3.125/0.59	3.056/0.53	0.434	Power	1	1.986	0.574	0.002
Offer	80	3.100/0.64	2.873/0.71	0.036	Interaction	6	23.575	0.000	0.113
Refusal	80	2.623/0.54	3.458/0.55	0.000					
Request	80	3.630/0.52	3.614/0.58	0.852					
Suggestion	80	3.735/0.54	3.027/0.52	0.000					
Thank	80	3.917/0.61	3.751/0.58	0.081					

### Rank of imposition

**Figure 4:**
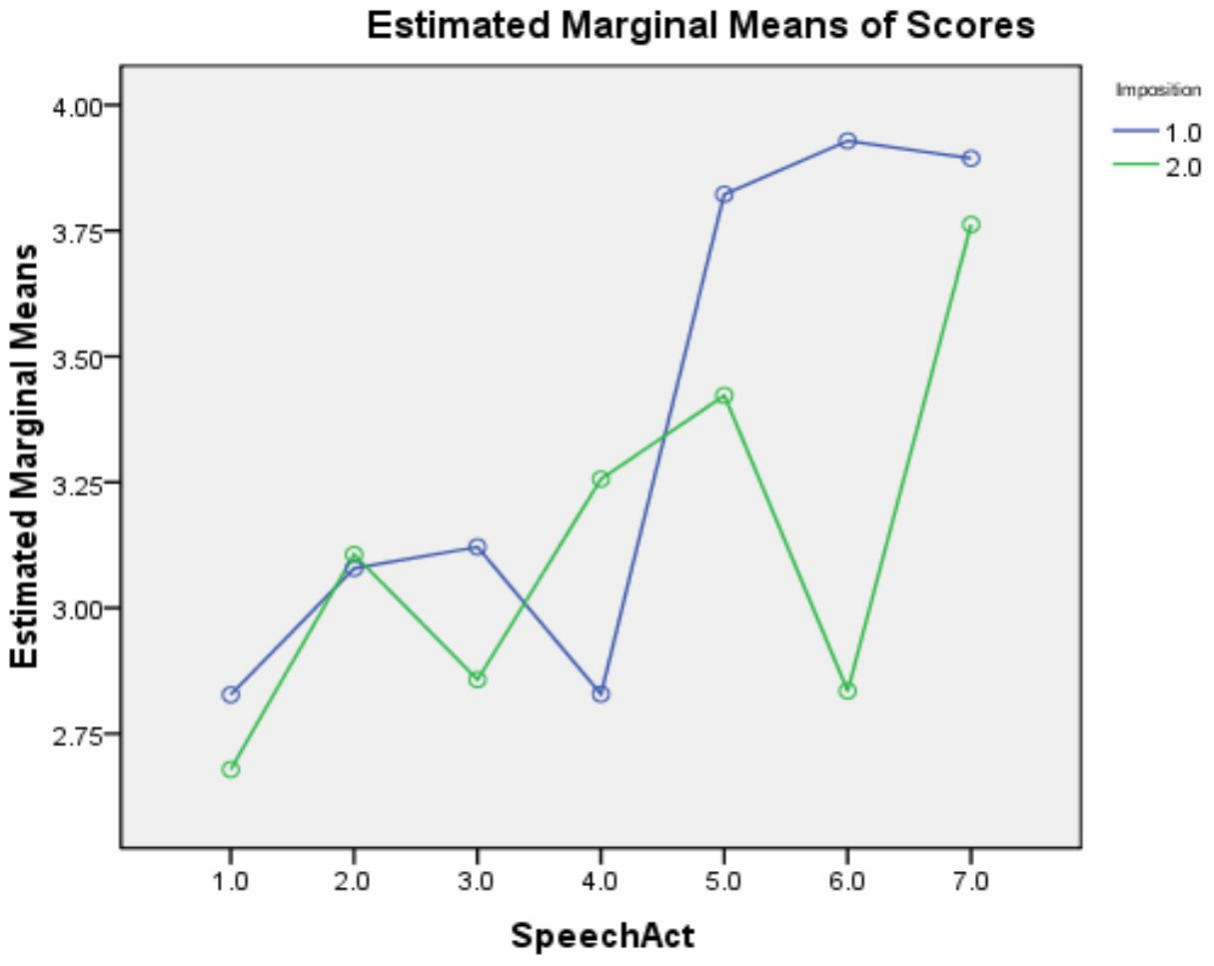
Profile plots for the interaction between speech act and rank of imposition. Speech act codes: 1 = Apology; 2 = Invitation; 3 = Offer; 4 = Refusal; 5 = Request; 6 = Suggestion; 7 = Thank. Imposition codes: 1 = R−; 2 = R+.

As shown in Table 4, the main effects of speech act [*F*(6,153) = 63.918, *p* = 0.000, η_p_^2^ = 0.257] and Imposition [*F*(6,153) = 39.300, *p* = 0.000, η_p_^2^ = 0.034] were both statistically significant, with large and small effect sizes, respectively. The interaction effect between the factors was also statistically significant with a medium effect size [*F*(6,153) = 23.635, *p* = 0.000, η_p_^2^ = 0.114]. Pairwise comparisons revealed that, compared with items with the R+ feature, those with the R-feature were significantly easier for Offer, Request, and Suggestion but significantly harder for Refusal. These results are also visualized in Figure 4.

**Table 4:** Comparison of mean task performance by speech act and rank of imposition.

Speech act	*N*	Mean/SD		Pairwise comparisons	Analysis of variance				
		R−	R+	*p*		df	*F*	*p*	η_p_^2^
Apology	80	2.827/0.84	2.679/0.57	0.193	Speech act	6	63.918	0.000	0.257
Invitation	80	3.079/0.69	3.106/0.53	0.763	Imposition	1	39.300	0.000	0.034
Offer	80	3.121/0.64	2.857/0.59	0.008	Interaction	6	23.635	0.000	0.114
Refusal	80	2.829/0.64	3.256/0.51	0.000					
Request	80	3.822/0.50	3.422/0.57	0.000					
Suggestion	80	3.928/0.53	2.835/0.53	0.000					
Thank	80	3.894/0.69	3.762/0.58	0.195					

## Discussion

ODCTs are a special type of oral assessment that elicit one-sided responses in hypothesized conversations. Following the suggestion by Fulcher and Reiter (2003), we included both pragmatic categories (i.e., the eight speech acts) and cultural factors (i.e., the combinations of the three social variables in different scenarios) in developing ODCT tasks in the current study. The analysis of the appropriateness ratings of our participants’ responses to the ODCT items revealed several substantive findings. First, the MFRM analysis showed that the eight speech acts investigated can be ranked in ascending order of difficulty for Chinese EFL learners as follows: Thank, Request, Suggestion, Disagreement, Invitation, Refusal, Offer, and Apology. Second, the two-way ANOVAs revealed significant main effects of speech act and rank of imposition (R), but not of power (P) and social distance (D). These analyses also revealed significant interaction effects between speech act and each of the three contextual variables, confirming the importance of including both pragmatic categories and cultural factors in ODCT task design (Fulcher and Reiter, 2003). We discuss our findings on the relative difficulty of the tasks for different speech acts and the interaction effects between speech act and the three contextual variables below.

### Difficulty of ODCTs for different speech acts

Previous findings on the relative difficulty of pragmatic tasks on different speech acts are limited and inconsistent. In testing learners’ pragmatic knowledge of three speech acts: apology, request, and refusal, Hudson (2001) found that apologies were slightly easier than requests and refusals for Japanese ESL learners, which was echoed by Roever’s pragmatics test of ESL/EFL learners with diverse language background (Roever, 2004). Hudson accounted for this difference with the explanation that apologies tended to be more formulaic than the other two speech acts and attributed the absence of other difficulty differences to the homogeneity of the participants’ proficiency level. Using data from Ahn (2005) on L1 English learners of Korean as a foreign language (KFL) at diverse proficiency levels, Brown (2008) and Brown and Ahn (2011) reported that the average ratings of apologies, requests, and refusals were comparable. Liu (2006), however, found apologies to be consistently more difficult across three test formats (MDCT, DSAT, and WDCT) than requests for Chinese EFL learners at diverse proficiency levels. The different findings pertaining to the difficulty of apologies relative to other speech acts on learners with different L1 backgrounds and the agreement between Liu’s finding and our finding that apologies were harder than requests for Chinese EFL learners suggest a potential effect of the learners’ L1 cultural background on speech act production task difficulty. This conclusion aligns with the prediction that the culture-specific nature of pragmatic ability may give rise to unique challenges for learning L2 speech acts (Taguchi, 2012). Youn and Brown’s (2013) finding that pragmatics test item difficulty remained consistent across two different studies by Ahn (2005) and Youn (2008) on two different groups of L1 English KFL learners also offers support for this conclusion, as it suggests more consistency of task difficulty among learners of the same L1 background.

Apology was found to be the most difficult speech act for Chinese EFL learners in the present study. A closer examination of the production data revealed that our participants had no difficulty in using the formulaic head act strategy (i.e., *I’m sorry*), but many struggled with producing appropriate supporting moves. As illustrated in Example 1, many students followed *I’m sorry* with an explanation that the cause was accidental, often with the structure “didn’t … on purpose”, likely translated from the Chinese expression *búshì gùyì de* (不是故意的, “didn’t do it on purpose”), which is commonly used in apologies in Chinese. This strategy, however, was not considered conventional by the L1 English raters.

**Table:** 

(1)	a. I’m sorry. I did it by accident.
	b. I’m so sorry. I did not do it on purpose. I promise it will not happen again.
	c. I’m so sorry. I did not knock over the cup on purpose.

In addition, some participants provided grounders that were considered by the L1 English raters to be too casual to the extent that they jeopardize the sincerity of the apology, as illustrated by Example 2:

**Table:** 

(2)	a. Sorry, Miss May, I had something important to do just now. So I’m coming late.
	b. Sorry, Miss May, I had something on the way. I’m very sorry.
	c. Sorry, I have something urgent. Please forgive me.

These grounders also appeared to display an L1 transfer effect, as the expressions *yǒudiǎn shì* (有点事, “have something”) and *yǒudiǎn jíshì* (有点急事, “have something urgent”) are commonly used excuses in apologies in Chinese. These examples support Blum-Kulka’s (1982) claim that L2 learners’ speech act production is often influenced by pragmatic transfer from their L1 and that negative transfer may result in pragmatic failures and cross-cultural communication breakdowns.

Offer was found to be the second most difficult speech act for Chinese EFL learners. Previous research on L2 learners’ realization of offers is scant. As offers have a directive nature in that they involve the speaker attempting to persuade the hearer to accept the offer in question, the use of head act strategies for offers resembles that for requests. However, a major difference between offers and requests is that offers presumably benefit the hearer while requests impose on the hearer. As such, the use of direct strategies may be considered more acceptable for offers than for requests, which is also the case in Chinese. Additionally, it has been noted that in some cultures, Chinese included, an offer is not considered sincere until it has been reiterated (Barron, 2003). As noted by the L1 English raters, the participants’ offers received low ratings primarily because they sometimes sounded overly direct and eager to help to the extent that the hearer might feel being imposed on. In Example 3, one participant offered to help a sick classmate with the use of *must*, which the raters felt was overly strong.

**Table:** 

(3)	You are sick. I must take you to the hospital.

Refusals were found to be the third most difficult among the eight speech acts. As a typical face-threatening speech act (Brown and Levinson, 1987), refusals have been recognized as a major cross-cultural obstacle (Babai Shishavan and Sharifian, 2016). Ekiert et al. (2018) reported that advanced L1 Japanese and Spanish ESL learners achieved comparable pragmatic appropriacy for refusals, complaints, and advice, but lower proficiency ESL learners with those L1 backgrounds achieved lower pragmatic appropriacy for refusals than for complaints and advice. Our results showed that refusals were harder than suggestions for Chinese EFL learners. Refusal was again found more difficult than most speech acts in the present study. Previous research found that grounder and regret strategies are the most frequently used for refusals by Greek foreign language learners (Bella, 2014) as well as by Chinese learners of English in both at-home and study aboard contexts (Ren, 2015). A close analysis of the participants’ production data indicated that they relied heavily on expressions of gratitude but rarely used empathetic or positive statements, as illustrated in the participant’s response to the item on refusing a chance to take part in a speech contest in Example 4. One L1 English rater commented that a positive statement before the refusal (e.g., *I know the speech contest is a great opportunity for me to practice my English*, *but…*) would improve its pragmatic appropriacy.

**Table:** 

(4)	I’m sorry. I do not think I can take part in it. Thank you for your trust.

Request, Suggestion, Disagreement, and Invitation were found to be relatively easier, and Thank was found to be the easiest speech act. The participants demonstrated good familiarity with the pragmatic formulas associated with these speech acts, and they used the most formulaic expressions for Thank among all speech acts. The higher frequency of use of these speech acts in the university setting in general and in the language classroom in particular may have also contributed to the lower difficulty of these speech acts.

### The interaction effects between speech act and The three contextual variables

The difficulty of the ODCT items was found to be affected by the interaction between speech act and each of the three contextual variables. This finding is consistent with Taguchi’s (2007) finding that social factors may make certain types of situations for pragmatic tasks more demanding than others. The finding also supports Fulcher and Reiter’s (2003) claim that different contextual variables may have distinct effects on particular speech acts.

Social distance exhibited different effects on different speech acts. Compared with items with the D+ feature, items with the D-feature were significantly easier for Offer and Request but significantly harder than Suggestion and Thank. These results indicate that the participants produced more appropriate offers and requests to familiar hearers but more appropriate suggestions and thanks to unfamiliar hearers. A close analysis of the learner production data suggested that the participants tended to use similar types of formulaic strategies for items with D+ and D− features. For example, they frequently used “Would you like to …” for Suggestion and “Thank you very much” for Thank, which were considered more appropriate for unfamiliar hearers (D+) but sometimes overly polite for very familiar peers (D−). Li (2010), for example, indicated that native Australian students tended to use ability statements such as “You can” to realize suggestions in D-scenarios.

With respect to power, items with the P-feature were significantly easier for Offer and Suggestion, while items with the P= feature were significantly easier for Refusal. These results indicate that the participants produced more appropriate offers and suggestions to hears with more power but more appropriate refusals to hears with equal power. These results may not be surprising, as they align with the common understanding that it is easier to make an offer to than to refuse someone with more power in the university setting (e.g., a teacher) in the Chinese culture. Overall, our participants demonstrated some struggle with consistently deploying politeness strategies appropriate for these speech acts to hearers with different power status, sometimes showing negative pragmatic transfer from Chinese. For example, they tended to extend offers to teachers using polite, indirect forms and to their peers using highly direct forms (e.g., *Come to dinner with me*). While such direct strategies for making offers to peers are commonly used to show sincerity and hospitality or to preserve the speaker’s positive face in the Chinese culture, they may sound intruding in western cultures where the hearer prefers to be left alone (Gu, 1990; Mao, 1994).

Imposition was the only contextual variable that showed a significant main effect, with items with the R+ feature showing a higher level of difficulty than those with the R− feature overall. Hudson (2001) and Liu (2006, 2007) also reported that R+ items received lower scores than R− items across multiple test methods, although they did not examine the interaction between speech act and imposition. Our analysis showed that, compared to R− items, R+ items were significantly harder for Offer, Request, and Suggestion, significantly easier for Refusal, and comparably difficult for other speech acts. While these findings are not necessarily surprising (e.g., as the degree of imposition increases, requests become harder while refusals become easier), they nonetheless provide evidence for the need and usefulness to look at the interaction effect between speech act and individual situational variables.

### Limitations

The current study has several limitations that can be addressed in future research. First, while we included participants with diverse levels of English proficiency in the study to have a heterogenous sample, we did not systematically examine the effect of proficiency on the difficulty of speech act production tasks, a topic that can be useful to investigate in future research. Second, our analysis focused on the appropriateness ratings of the participants’ responses only, and it may be useful for future research to consider learners’ perceptions of task difficulty and to qualitatively explore the reasons why learners see certain speech acts and contextual variable combinations are more difficulty than others. Third, we employed two raters in the current study only, and greater reliability in the judgments of language learners’ pragmatic performance could be achieved by using a larger pool of raters. Fourth, a certain degree of interference existed in the data collection phase as oral samples of a group of participants were elicited simultaneously in a language lab, which can be avoided by applying headphones or collecting data separately. Finally, given that the difficulty of oral speech act production tasks may vary by L1 cultural background, the order of relative difficulty established in the current study for the eight speech acts may not be directly applicable to English learners of other L1 backgrounds. Future research can investigate how the order of relative difficulty may vary by L1 background by including participants from diverse L1 backgrounds.

## Conclusion

This study examined the relative difficulty of oral speech act production tasks involving eight types of speech acts for Chinese EFL learners and the effects of three situational variables, namely, power, social distance, and imposition, on such difficulty. A Many-facet Rasch Measurement analysis suggested that the eight speech acts can be ordered by ascending difficulty as follows: Thank, Request, Suggestion, Disagreement, Invitation, Refusal, Offer, and Apology. Significant effects on performance scores were found for the interaction between each of the three contextual variables and speech act, and the specific effects observed varied by speech act. Learner responses also reflected influences of their L1 cultural background. Our findings on the relative difficulty of oral production tasks involving different speech acts and the effects of relevant situational variables on such difficulty have useful implications for L2 pragmatics test design.

Our findings have useful implications for L2 pragmatics testing. Given that different speech act types are not equally difficult to EFL learners, it is important to not generalize results from testing the realization of a particular speech act or a small set of speech acts to the learners’ pragmatic ability in performing other speech acts. Furthermore, given the effects of the situational variables on the task difficulty for different speech acts, it is critical to test learners’ speech act production with different combinations of contextual variables. Finally, the evaluation of task difficulty in L2 pragmatics assessment need to take learners’ L1 background into account.

Our findings also have useful implications for L2 pragmatics pedagogy in the Chinese EFL context. From a task-based language teaching perspective, as advocated by Taguchi and Kim (2018), the relative difficulty of tasks provides highly useful information for task selection and task sequencing in teaching L2 pragmatics. The rank of difficulty estimates of the pragmatic tasks for different speech acts observed in the present study can be used to inform the order in which the speech acts are introduced and the allocation of classroom time to different speech acts in L2 pragmatics pedagogy. Our findings regarding the effects of the three contextual factors on the task difficulty for different speech acts can be used to inform the design of different situation types in teaching speech acts. Our findings further showed the need to help Chinese EFL learners become more sensitive to different situation types and to avoid negative L1 transfer in their choices of speech act strategies. To this end, it will be especially helpful to deploy learning activities designed to help learners become more aware of the pragmatic appropriacy of different speech act strategies in different situation types as well as differences between the pragmatic appropriacy of different speech act realizations in the learners’ L1 and the target language.

## Data availability statement

The original contributions presented in the study are included in the article/Supplementary material, further inquiries can be directed to the corresponding author.

## Author contributions

All authors listed have made a substantial, direct, and intellectual contribution to the work and approved it for publication.

## Conflict of interest

The authors declare that the research was conducted in the absence of any commercial or financial relationships that could be construed as a potential conflict of interest.

## Publisher’s note

All claims expressed in this article are solely those of the authors and do not necessarily represent those of their affiliated organizations, or those of the publisher, the editors and the reviewers. Any product that may be evaluated in this article, or claim that may be made by its manufacturer, is not guaranteed or endorsed by the publisher.

## Supplementary material

The Supplementary material for this article can be found online at: https://www.frontiersin.org/articles/10.3389/fpsyg.2023.1096399/full#supplementary-material
