# Supplementary material for: The difficulty of oral speech act production tasks in second language pragmatics testing
Source: Front Psychol. 2023 Feb 3;14:1096399. doi: 10.3389/fpsyg.2023.1096399 (PMC9937551; doi:10.3389/fpsyg.2023.1096399)
Supplement: Supplementary file 1 [file Table_1.DOCX]

**Appendix. Oral Discourse Completion Tasks Used**

Note: The item numbers at the end of the items correspond to the eight item types listed in Table 1. The time limit was 20 seconds for the first 50 items and 50 seconds for the last 10 items.

Directions: Please read each scenario description on the screen and speak out your response within the time limit specified for that scenario.

Example:

You ask a teacher in the office which desk is your teacher Miss May’s.

You might say:

Excuse me, Miss, sorry to bother you, but do you know which desk is Miss May’s?

Now let’s begin the test.

1. Your classmate Mary is going to the cafeteria for lunch. Ask her to save a seat for you. (Request item 1)
2. Someone is asking for directions to Teaching Building No. 2. Offer to show the stranger the way. (Offer item 2)
3. Someone asks you how to look for a book in the library. You suggest that he/she go to the librarian for help. (Suggestion item 2)
4. You are planning John’s birthday party on Saturday. Invite his classmate Mary to the party. (Invitation item 1)
5. Your roommate Mary brings lunch for you. You thank her. (Thank item 1)
6. You accidentally step on a classmate (Matt)’s foot. Apologize to him. (Apology item 1)
7. A friend invites you to eat out. Tell him/her you can’t go. (Refusal item 1)
8. You watched a movie with your friend Paul. He thinks it’s a great movie, but you disagree. (Disagreement item 1)
9. Ask a stranger in the dorm next-door to turn down his/her music. (Request item 2)
10. Offer to help your roommate Mary pick up a package (取包裹) on your way to the cafeteria. (Offer item 1)
11. Your roommate Mary is feeling bored. You suggest that she watch a movie. (Suggestion item 1)
12. Invite a new classmate Lily to have lunch with you at the cafeteria. (Invitation item 2)
13. You forget to bring a pen to the library. Someone sitting next to you notices and offers you one. Thank him/her. (Thank item 2)
14. You accidentally bump into (撞上) a student whom you do not know and the books he is carrying fall onto the ground. You stop, pick up the books, and apologize. (Apology item 2)
15. Some student that you don’t know well asks you to go to dinner together. You refuse. (Refusal item 2)
16. You meet Matt for the first time at a friend’s party. You are talking about part-time jobs. He thinks it’s beneficial for college students, but you disagree. (Disagreement item 2)
17. You are attending Miss May’s class and you have difficulty following some point. You ask her to clarify it again. (Request item 3)
18. Offer to carry a bundle of exercise books (一捆练习本) for your teacher (Miss May). (Offer item 3)
19. You are in a class. You suggest that your teacher Miss White make the words on the PPT larger. (Suggestion item 3)
20. Invite your teacher Miss May to dinner tomorrow. (Invitation item 3)
21. Your teacher Mr. Brown explains some difficult points to you during the break. You thank him. (Thank item 3)
22. You are a few minutes late for your 9am appointment with your teacher Miss May in her. You apologize. (Apology item 3)
23. Your teacher Miss White suggest that you take part in a speech contest. But you don’t want to try. (Refusal item 3)
24. Your teacher Miss May joins you in your group discussion and expresses an opinion. You disagree. (Disagreement item 3)
25. Professor Smith delivers an invited lecture at your university. You ask him for his PPT slides (幻灯片) after his lecture. (Request item 4)
26. Offer to share an umbrella with an unfamiliar teacher on a rainy day. (Offer item 4)
27. It’s very hot. You suggest that a librarian set the AC to a lower temperature. (Suggestion item 4)
28. Invite a teacher Mr. Chris whom you don’t know to be a judge for a speech contest in your class. (Invitation item 4)
29. You ask a teacher in the office for the location of a professor’s office. She shows you the way. You thank her. (Thank item 4)
30. You are attending a lecture. You accidentally knock over a cup on the desk and spill water over the notes of a teacher whom you do not know. You apologize. (Apology item 4)
31. Mr. Black who has not taught you before asks you to share your travel experiences in his class. You don’t want to go. (Refusal item 4)
32. You and your close friend Peter are members of the Student Union. You are attending a meeting to discuss candidates for the President. Peter suggests Jay, but you disagree. (Disagreement item 5)
33. Ask your close friend Mike to lend you some money for next month’s expenses. (Request item 5)
34. Offer to take your sick roommate Jill to the hospital. (Offer item 5)
35. Given your friend (Sue)’s poor academic performance, you suggest that she change her major. (Suggestion item 5)
36. You are organizing a New Year’s party for your class. Your friend Peter is a good singer. Invite him to come to the party to sing a song. (Invitation item 5)
37. Your friend Mike throw you a successful surprise party for your birthday. You thank him. (Thank item 5)
38. You borrow a friend’s laptop, but you accidentally knock it off the table and it falls and breaks. You apologize to your friend (Mike). (Apology item 5)
39. Your close friend John asks you to lend him some money for next month’s expenses, but you can’t. (Refusal item 5)
40. You know your teacher Professor Lee has been very busy. You ask him to spare an hour to discuss your final paper with you. (Request item 7)
41. You see someone slip (滑倒) and his leg is bleeding. You offer to take him to the clinic for a check-up. (Offer item 6)
42. You are attending a discussion group with students from other universities. The speaker is asking for feedback (反馈). You suggest that he try a different method. (Suggestion item 6)
43. As Chairman of the Students’ Union, you invite your teacher Miss May to attend the College’s Christmas party this weekend. (Invitation item 7)
44. In your senior year, you are looking for a job. Your teacher Miss White recommends (推荐) you for a position with a competitive salary. You thank her. (Thank item 7)
45. You are attending a symposium (研讨会). A fellow student offers good suggestions for your paper. You thank him. (Thank item 6)
46. Andrew lives in the dorm next door. You two are not close. He asks you to lend him your laptop for a day. You don’t want to. (Refusal item 6)
47. You are not familiar with Professor Lee. As Chairman of the Students’ Union, you invite him to the College’s New Year party. (Invitation item 8)
48. Your teacher Miss May is planning a meeting. You offer to help organize it. (Offer item 7)
49. In an interview, thank the interviewer for giving you the chance to be interviewed. (Thank item 8)
50. Your teacher Mr. Brown is asking for feedback about his class. You suggest that he integrates more discussions in class. (Suggestion item 7)
51. You are at the library. You accidentally knock over a cup which falls and breaks into pieces. The cup belongs to a student who sits next to you. You don’t know the student. You apologize to him/her. (Apology item 6)
52. You are playing soccer on the soccer field. You take a shot and the ball hits a teacher on the back of his/her head very hard. You go up to the teacher and apologize. (Apology item 8)
53. You are waiting for the bus home. Your teacher Miss May offers you a ride home. You don’t want to cause her any trouble and decline. (Refusal item 7)
54. You are working as an intern (实习生) in a company. You ask your boss Mr. Smith for a 5-day leave in the finals week. (Request item 8)
55. A manager Mr. Smith who interviewed you calls to tell you that you are hired, but you don’t want the job any more. (Refusal item 8)
56. You are organizing a debate and you learn that Matt is a member of the school’s debate team (校辩论队一员). You are meeting him for the first time, and you invite him to be a judge for the debate. (Invitation item 6)
57. You are writing a paper that is due tomorrow on the computer at midnight. The computer breaks down, and you learn that Jay who lives next door is a computer expert. You’ve never met him in person, but you decide to ask him for help even though it is late. (Request item 6)
58. Your adviser Professor Black is very busy, and he works hard to arrange a meeting with you this afternoon to discuss your thesis with you. You receive a phone call for a job interview. You really want this job and decide to go to the interview instead of meeting with the professor. Apologize to him. (Apology item 7)
59. You hear from your roommate Robin that his teacher Miss White’s computer has broken down. You are a computer major. You offer to help repair the computer for Miss White who doesn’t know you. (Offer item 8)
60. Your friend Mary and her parents don’t get along. Her mother Mrs. Lee comes to see you and ask you for advice. You suggest that she plan a trip with Mary this weekend to improve things. (Suggestion item 8)
